# Intravenous Contrast Medium Administration for Computed Tomography Scan in Emergency: A Possible Cause of Contrast-Induced Nephropathy

**DOI:** 10.1155/2015/805786

**Published:** 2015-10-20

**Authors:** Lantam Sonhaye, Bérésa Kolou, Mazamaesso Tchaou, Abdoulatif Amadou, Kouméabalo Assih, Bidamin N'Timon, Kokou Adambounou, Lama Agoda-Koussema, Komlavi Adjenou, Koffi N'Dakena

**Affiliations:** ^1^Department of Radiology, University Teaching Hospital of Lomé, Lomé, Togo; ^2^Department of Radiology, University Teaching Hospital of Kara, Kara, Togo

## Abstract

The goal of this study was to assess risk for CIN after CT Scan during an emergency and to identify risk factors for the patient. Prospective review of all patients admitted to the emergency room (ER) of the Teaching Hospital of Lomé (Togo) during a 2-year period. CIN was defined as an increase in serum creatinine by 0.5 mg/dL from admission after undergoing CT Scan with intravenous contrast. A total of 620 patients underwent a CT Scan in the emergency room using intravenous contrast and 672 patients took the CT Scan without intravenous contrast. Out of the patients who received intravenous contrast for CT Scan, three percent of them developed CIN during their admission. Moreover, upon discharge no patient had continued renal impairment. No patient required dialysis during their admission. The multivariate analysis of all patients who had serial creatinine levels (including those who did not receive any contrast load) shows no increased risk for acute kidney injury associated intravenous contrast (odds ratio = 0.619, *p* value = 0.886); only diabetes remains independent risk factor of acute kidney injury (odds ratio = 6.26, *p* value = 0.031).

## 1. Introduction

Contrast-induced nephropathy or contrast medium-induced nephropathy (CIN), defined as an increase in serum creatinine (SCreat) greater than 25% or ≥ 0.5 mg/dL within three days of intravenous (IV) contrast administration in the absence of an alternative cause, is the third most common cause of new acute renal failure in hospitalized patients [1].

The association between the administration of IV contrast media (CM) and the development of acute kidney injury has been well documented.

The European Society of Urogenital Radiology (ESUR) described the risk factors for CIN, which are

- patient-related: estimated glomerular filtration rate (eGFR) less than 60 mL/min/1.73 m^2^ before intra-arterial administration, eGFR less than 45 mL/min/1.73 m^2^ before intravenous administration, in particular in combination with diabetic nephropathy, dehydration, congestive heart failure, recent myocardial infarction (<24 h), intra-aortic balloon pump, periprocedural hypotension, low hematocrit level, age over 70, concurrent administration of nephrotoxic drugs, and known or suspected acute renal failure;
- procedure-related: intra-arterial administration of contrast medium, high osmolality agents, large doses of contrast medium, and multiple contrast medium administrations within a few days [2].

Contrast media (CM) are increasingly used in diagnostic and interventional procedures. This results in the rising incidence of iatrogenic renal function impairment caused by the exposure to CM, a condition known as CIN. Radiographic CM are responsible for 11% of cases of hospital-acquired renal insufficiency, the third most common cause of renal failure after impaired renal perfusion and the use of nephrotoxic medications [1]. Among all procedures utilizing CM for diagnostic or therapeutic purposes, coronary angiography and percutaneous coronary interventions (PCI) are associated with the highest rates of CIN [1].

This incidence applies to contrast-enhanced CT which is estimated to be 4.96% even if it varied based on the presence of various risk factors [3, 4].

The goal of this study was to assess the risk for CIN after CT Scan in emergency, to identify patient risk factors and to evaluate the rate of CIN in high-risk subgroups.

## 2. Materials and Methods

This was a prospective study of all patients admitted during a two-year period (January 1, 2012, to December 31, 2013) for a CT Scan in emergency at Lomé (TOGO) University Hospital.

All patients who underwent initial diagnostic management using IV contrast for CT Scan of brain, of the chest and of the abdomen and pelvis, were included in this study. Patients with a known history of end stage renal disease or with no follow-up serum creatinine levels after contrast dose were excluded.

For all patients we recorded age; sex; serum creatinine levels; systolic blood pressure (SBP); and preadmission medical comorbidities, including diabetes and renal insufficiency.

We also studied two high-risk subgroups: patients with diabetes who underwent CT scanning in emergency and patients with renal impairment (SCreat > 1.5 mg/dL) on admission who underwent CT scanning in emergency.

All patients in this study were hemodynamically stable.

The bolus of iomeprol 350 (low-osmolar, nonionic iodinate) IV contrast delivered before the brain, chest, abdomen, and pelvis portion of the CT scans depending on the weight of the patient: patients weighing > 95 kg receive 150 mL; patients weighing 81–95 kg received 120 mL; and patients weighing less than 81 kg receive 1.5 mL/kg.

We defined CIN as an increase in serum creatinine (SCreat) ≥ 0.5 mg/dL from the baseline [3] and renal insufficiency when SCreat > 1.5 mg/dL on admission.

We analyzed the results using descriptive statistics software SPSS20.0. Chi-square analysis was used to evaluate the proportion of patients that developed CIN after the use of IV contrast. *p* values of less than 0.050 were considered significant. Variables were included in the multivariate analysis if they were identified as risk factors associated with CIN in univariate analysis (*p* < 0.050).

## 3. Results

A total of 2780 patients underwent CT scan in emergency during the study. CT scan using IV contrast was given to 1068 patients (38.4%). 448 patients were excluded from the study due to insufficient levels of serial serum creatinine available and preexisting and-stage renal disease requiring long-term hemodialysis. On the 1712 patients (61.6%) who underwent a CT Scan without IV contrast, 672 patients (with serial serum creatinine) were also analyzed. These patients did not have contraindication for IV contrast Scan; they did not have CT Scan with IV contrast, and, because of that, the patients did not necessitate contrast evaluation (Table 1).

The median age of the patients undergoing CT Scan with IV contrast was 51 years; the median age undergoing CT Scan without contrast was 57 years (Table 2).

All of the patients who developed CIN returned to baseline renal function; no patient required dialysis.

The patients who developed CIN were more likely to be diabetic (Table 3).

The multivariate analysis using a binary logistic regression found that being male patient (adjusted odds ratio = 1.23 and *p* value = 0.349), age (adjusted odds ratio = 1.91 and *p* value = 0.039), admission systolic blood pressure (adjusted odds ratio = 1.13 and *p* value = 0.923), renal insufficiency (adjusted odds ratio = 1.79 and *p* value = 0.517), and dose of IV contrast (adjusted odds ratio = 1.14 and *p* value = 0.099) were not independently associated with increased risk for developing CIN. Only preexisting diabetes is independently associated with increased risk for developing CIN (adjusted odds ratio = 7.21 and *p* value = 0.019).

The multivariate analysis of all patients who had serial creatinine levels (including those who did not receive any contrast load) shows no increased risk for acute kidney injury associated intravenous contrast (odds ratio = 0.619, *p* value = 0.886); only diabetes remains independent risk factor of acute kidney injury (odds ratio = 6.26, *p* value = 0.031).

Four (7.8%) of 51 renal insufficiency patients developed CIN during their admission; but no patient were discharged with continued CIN. This rate of CIN is higher than those of the general population studied (3%), but this difference was not significant (*p* value = 0.495). No other patient characteristics were associated with development of CIN in the patients with renal insufficiency on admission apart from their tendency to be older (median age 66 years versus 53 years; *p* value = 0.021).

Eight (22.2%) of the 36 diabetic patients developed CIN during their admission; no diabetic patient discharged had continued CIN. Only four of the diabetic patients had renal insufficiency on admission. No other patient characteristics were associated with the development of CIN in this study group.

## 4. Discussion

The intravenous contrast before Computed Tomography scanning help to identify injuries and better treat patients. In the literature, the contrast-induced nephropathy (CIN) has been studied extensively in patient's followings coronary angiography [5, 6]. There are few studies evaluating CIN in emergency and they are limited studies evaluating CIN in emergency [7].

The exact mechanism of leading to CIN is not clear, but combinations of toxic and ischemic injury to tubular cells are suggested as contributing factors. Increased fluid viscosity secondary to the contrast agent concentration due to medullar hyperosmolar environment, which leads to decreased flow in the medullary tubules and vessels, is proposed to be one of the mechanisms for CIN [8, 9]. Direct cytotoxic effect of contrast medium on tubular cells is also one of the mechanisms suspected to cause tubular cells injury [8].

The risk factors are likely to contribute to an increase in kidney injury [10].

The incidence of CIN is up to 12% in patients who underwent CT Scan of the chest with intravenous contrast [11, 12]. In our study, the incidence of CIN of 3% in emergency CT Scan was reported. No patient had abnormal serum creatinine levels upon discharge. The incidence of acute kidney injury (AKI) of various and unknown origin in these patients is low. In the present clinical setting, it does not support the premise that contrast media administration induced AKI.

This study is similar to other studies with rang of CIN rates from 1.9% to 6.6% [13, 14].

For instance, our study found that renal insufficiency, diabetes, age older than 55 years, and intravenous contrast dos of more than 150 mL were all risk factors of CIN in univariate analysis, similar results were found in the study of Colling et al. [7], and thus our study shows that only diabetes is found to be independently associated with an increased risk for CIN in multivariate analysis.

Likewise, for patients receiving contrast for other reasons, the risk factors are similar to those identified in other studies [7, 13, 14].

Diabetes does seem to increase the risk for CIN in most patients. Diabetes remains an independent risk factor of development of CIN in most studies [4, 15]. Our study came to the same conclusion.

In our study, we did not show an association between renal insufficiency on admission and increase risk for CIN. Colling et al. [7] in their study reached a similar conclusion. However, this must be confirmed in large group size study.

This study also reported no difference in rates of acute kidney injury in patients undergoing CT Scan with contrast and those undergoing a CT Scan with contrast. Contrary to what Colling et al. [7] reported in their study, the number of patients who did not undergo CT Scan with IV contrast and also had measured serial creatinine levels was large (672 patients). However, no difference in rates of acute kidney injury in patients undergoing CT Scan with contrast and those undergoing a CT Scan with contrast has been reported in the studies of Matsushima et al. [16] and McDonald et al. [17].

## 5. Conclusion

The incidence of CIN after CT Scan with intravenous contrast in emergency is 3%. Diabetes is only independent risk factor associated with CIN in our multivariate analysis. So, in emergency, the CT Scan with intravenous contrast must be used if that did necessitate contrast evaluation to identify injuries and better treat patients.

## Figures and Tables

**Table 1:** Patient selection for the study.

	Number (%)
CT^*∗*^ Scan in emergency	2780 (100%)
CT Scan in emergency with IV^*∗∗*^ contrast	1068 (38.4%)
CT Scan in emergency without IV contrast	1712 (61.6%)
CT Scan in emergency with IV contrast and serial creatinine levels	620 (22.3%)
CT Scan in emergency without IV contrast and with serial creatinine levels	672 (24.2%)

CT^*∗*^: Computed Tomography; IV^*∗∗*^: intravenous.

**Table 2:** The demographic data of patients who underwent Computed Tomography Scan with intravenous contrast and those who did not receive intravenous contrast in emergency.

	All patients *N* = 1292	CT^*∗*^ with IV^*∗∗*^ contrast *N* = 620	CT without IV contrast *N* = 672	*p* value
Age, median (range)	53 (1–79)	51 (1–79)	57 (3–73)	0.283
Sex (male/female)	721/571	369/251	352/320	0.497
Admission serum creatinine	1.0	1.0	1.0	0.311
Highest serum creatinine	1.0	1.0	1.0	0.396
Serum creatinine at discharge	1.0	1.0	0.9	0.477
Diabetes	65 (5%)	36 (6%)	29 (4%)	0.419
Admission SBP^*∗∗∗*^	124	125	128	0.217
AKI^*∗∗∗∗*^ during admission	33 (3%)	21 (3%)	12 (2%)	0.741
AKI on discharge	0 (0%)	0 (0%)	0 (0%)	—

CT^*∗*^: Computed Tomography; IV^*∗∗*^: intravenous; SBP^*∗∗∗*^: systolic blood pressure; AKI^*∗∗∗∗*^: acute kidney injury.

**Table 3:** The risk factors of development of contrast-induced nephropathy in univariate analysis.

	Number	Incidence of CIN^*∗*^ %	*p* value
Age			0.023
≥55 years old	193	7.8	
<55 years old	427	1.4	
Sex			0.719
Male	369	3.0	
Female	251	4.0	
History of diabetes			0.011
Yes	36	22.2	
No	584	2.2	
Admission systolic blood pressure			0.329
≥90 mmHg	601	3.3	
<90 mmHg	19	5.2	
Admission serum creatinine level			0.495
≥1.5 mg/dL	51	7.8	
<1.5 mg/dL	569	3.0	
Dose of intravenous contrast			0.071
≥150 mL	46	6.5	
<150 mL	574	3.1	

CIN^*∗*^: contrast-induced nephropathy.
